# A Pathway to High-Quality Heteroepitaxial Ga_2_O_3_ Films via Metalorganic Chemical Vapor Deposition

**DOI:** 10.3390/mi16121363

**Published:** 2025-11-29

**Authors:** Yifan Li, Yachao Zhang, Kelin Wang, Guoliang Peng, Shengrui Xu, Qian Feng, Jinbang Ma, Yixin Yao, Yue Hao, Jincheng Zhang

**Affiliations:** State Key Discipline Laboratory of Wide Band Gap Semiconductor Technology, School of Microelectronics, Xidian University, Xi’an 710071, China

**Keywords:** MOCVD, Ga_2_O_3_, Solid Film

## Abstract

This work systematically investigates the heteroepitaxial growth of β-Ga_2_O_3_ thin films under varied substrate and temperature conditions via metalorganic chemical vapor deposition (MOCVD). Comprehensive characterization reveals that both the substrate type and growth temperature significantly influence the crystalline quality, surface morphology, chemical composition, and defect structure. Films grown at higher temperatures generally exhibit superior crystallinity and closer-to-stoichiometry composition, and thus suggest a reduction in oxygen deficiency. Certain substrates are shown to facilitate high-quality epitaxial growth with smooth surfaces and excellent crystallographic alignment. These findings offer key insights into optimizing growth parameters for high-performance β-Ga_2_O_3_-based devices.

## 1. Introduction

Beta-gallium oxide (β-Ga_2_O_3_) is an emerging ultra-wide-bandgap (~4.9 eV) semiconductor with a high critical electric field (~8 MV/cm) and excellent thermal and chemical stability [1,2,3,4,5]. These properties make it a promising candidate for high-power electronic devices, solar-blind photodetectors, and extreme-environment applications [6,7,8,9,10]. However, the development of β-Ga_2_O_3_-based technology is hampered by the high cost and small wafer size of commercially available native substrates, driving significant interest in heteroepitaxial growth on compatible foreign substrates [11,12,13,14,15].

Heteroepitaxy of β-Ga_2_O_3_, however, faces fundamental challenges, primarily due to lattice and thermal expansion mismatch between the film and substrate. These mismatches often lead to high defect densities, residual stress, and degraded electronic performance. In particular, oxygen vacancies—the dominant native point defect in β-Ga_2_O_3_—play a decisive role in controlling electrical conductivity, affecting device performance and stability [16,17,18]. Therefore, understanding and controlling the formation of oxygen vacancies during heteroepitaxy is crucial for tailoring the material’s electronic properties.

To address these challenges, this work systematically investigates the heteroepitaxial growth of β-Ga_2_O_3_ films on five different substrates—sapphire with 0°, 1.5°, and 6° off-cut angles, p-GaN/sapphire, and AlN/sapphire—at two representative temperatures (400 °C and 800 °C) using MOCVD. The influence of substrate type and growth temperature on the crystal structure, surface morphology, stress state, oxygen non-stoichiometry, and defect content is thoroughly examined using high-resolution X-ray diffraction (XRD), atomic force microscopy (AFM), scanning electron microscopy (SEM), X-ray photoelectron spectroscopy (XPS), energy-dispersive X-ray spectroscopy (EDX), and Raman spectroscopy. The findings provide clear guidelines for selecting optimal heteroepitaxial conditions to achieve high-quality β-Ga_2_O_3_ films with controlled oxygen deficiency for future electronic and optoelectronic applications.

## 2. Experimental Section

This study systematically investigates the heteroepitaxial growth of β-Ga_2_O_3_ films by metalorganic chemical vapor deposition (MOCVD) under varied conditions. Two sets of experiments were conducted at growth temperatures of 800 °C and 400 °C, respectively. For each temperature, five different substrates were employed, resulting in a total of ten samples. The substrates used include: c-plane sapphire without off-cut, c-plane sapphire with a 1.5° off-cut, c-plane sapphire with a 6° off-cut, p-GaN on sapphire, and AlN on sapphire. For simplicity, the latter two are referred to as pGaN and AlN substrates throughout this paper. The p-GaN/sapphire and AlN/sapphire templates were commercially sourced. The p-GaN template has a nominal thickness of ~4 µm, a root-mean-square (RMS) surface roughness of <1 nm (over a 5 × 5 µm^2^ area), and a (0002) XRD rocking curve full width at half maximum (FWHM) of ~250 arcsec. The AlN/sapphire template has a nominal thickness of ~500 nm, an RMS roughness of <0.5 nm, and a (0002) rocking curve FWHM of ~150 arcsec. These characteristics confirm the good crystalline quality and smooth surfaces of the templates, which are critical parameters for the subsequent heteroepitaxial growth of Ga_2_O_3_ films.

Prior to growth, each substrate underwent a standard cleaning procedure to remove organic contaminants and minimize unintentional surface oxidation. This involved sequential ultrasonic cleaning in acetone, ethanol, and deionized water. Subsequently, the substrate was loaded into a homemade vertical oxide MOCVD system. To further ensure a clean and well-prepared surface, a thermal annealing step was performed at 900 °C for 5 min under an oxygen atmosphere. Following this pretreatment, the temperature was lowered to the target growth temperature of either 800 °C or 400 °C to deposit β-Ga_2_O_3_ films with a thickness of approximately 380 ± 30 nm. It is important to note that the growth rate may vary across different substrates due to effects related to interfacial stress and lattice mismatch. Triethylgallium (TEGa) and high-purity O_2_ were used as the gallium and oxygen precursors, respectively, with flow rates maintained at 50 sccm for TEGa and 2000 sccm for O_2_. The chamber pressure was kept constant at 50 Torr during deposition. After film growth, a post-growth annealing treatment was carried out at 950 °C for 5 min in a high-purity oxygen environment to enhance film quality.

The structural properties and crystalline quality of the as-grown β-Ga_2_O_3_ films were characterized by high-resolution X-ray diffraction (HRXRD) using a BRUKER D8 ADVANCE diffractometer (Bruker Corporation, Billerica, MA, USA). Surface morphology and roughness were examined by atomic force microscopy (AFM, Bruker Icon 3, Bruker Corporation, Billerica, MA, USA) and scanning electron microscopy (SEM, Helios 650, FEI, Hillsboro, OR, USA). The chemical composition and bonding states were analyzed by X-ray photoelectron spectroscopy (XPS, Thermo Scientific ESCALAB 250Xi, Thermo Fisher Scientific, Waltham, MA, USA). Furthermore, Raman spectroscopy (WITec alpha300 RS, WITec GmbH, Ulm, Germany) with a 532 nm laser excitation source was employed to gain further insight into the crystal structure and phase identification.

## 3. Results and Discussion

The growth temperature plays a critical role in determining the epitaxial growth mode and the resulting microstructural characteristics of the β-Ga_2_O_3_ films.

**Figure 1:**
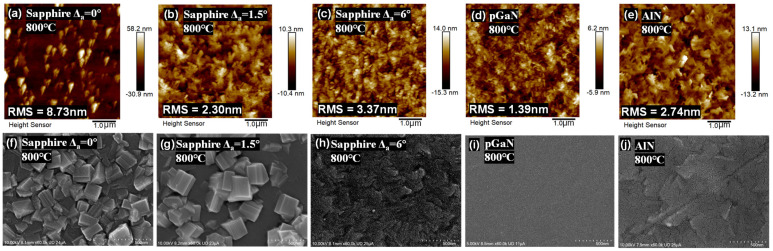
Surface morphology of β-Ga_2_O_3_ films heteroepitaxially grown on various substrates at 800 °C: (**a**–**e**) AFM top-view images (5 × 5 μm^2^ scan areas), and (**f**–**j**) corresponding SEM images (scale bar: 500 nm).

Figure 1 presents the surface morphology of the epitaxial films grown at 800 °C, investigated using both AFM and SEM measurements. At a higher growth temperature of 800 °C, the adatoms possess high surface migration energy. This elevated temperature ensures the complete decomposition of metalorganic precursors and facilitates the effective incorporation of oxygen species into the growing lattice. Consequently, the adatoms can migrate over long distances to find energetically favorable lattice sites, promoting two-dimensional, layer-by-layer growth.

This effect is corroborated by AFM (5 × 5 μm^2^ scan) and SEM images (with a 500 nm scale bar), which reveal the direct impact of substrate-film lattice mismatch on the macroscopic grain structure and surface morphology [19,20]. For instance, the film grown on c-plane sapphire without an off-cut angle exhibits significantly larger surface fluctuations. Its root mean square (RMS) roughness and the peak-to-valley height difference (as indicated in the AFM images) are approximately 4–5 times greater than those of films grown on other substrates under the same conditions. The underlying reason for this pronounced roughness will be discussed in a subsequent section.

The SEM images of films grown on sapphire substrates with different off-cut angles provide further insight. The film on the 6° off-cut sapphire demonstrates a relatively smooth surface with nearly continuous features. In contrast, films on 0° and 1.5° off-cut substrates display prominent, isolated protrusions. These features resemble monoclinic β-Ga_2_O_3_ aggregates with similar shapes and orientations, suggesting island-like growth. The superior morphology of the film on the 6° off-cut substrate implies that its surface provides more favorable atomic steps, conducive to the ideal growth of β-Ga_2_O_3_ [21].

Similarly, the film grown on the AlN template also exhibits a comparable, smooth morphology. Notably, the AFM and SEM results for the film on the pGaN template reveal the visually smoothest surface among all samples [22,23]. However, a comprehensive assessment of its crystalline quality requires further analysis in the following sections.

**Figure 2:**
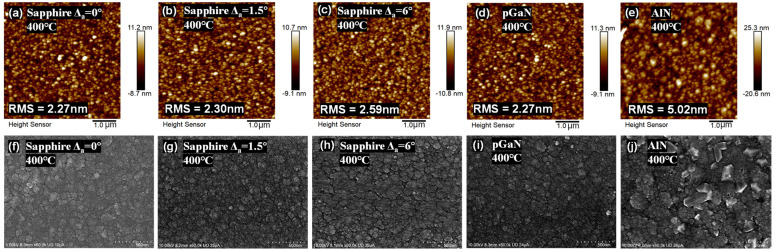
Surface morphology of β-Ga_2_O_3_ films heteroepitaxially grown on various substrates at 400 °C: (**a**–**e**) AFM top-view images (5 × 5 μm^2^ scan areas), and (**f**–**j**) corresponding SEM images (scale bar: 500 nm).

When the growth temperature is lowered to 400 °C, the situation changes markedly. Figure 2 presents the corresponding surface morphology of the films grown at 400 °C, as examined by AFM and SEM. The adsorbed molecules have substantially reduced migration energy due to the limited thermal budget. They tend to become immobilized before reaching the lowest-energy lattice sites, favoring a three-dimensional, island-like (Volmer-Weber) growth mode. Furthermore, the insufficient thermal energy may lead to incomplete decomposition of the metalorganic precursors and/or ineffective incorporation of oxygen species into the crystal lattice. As evidenced by both AFM and SEM, all five samples grown at this temperature exhibit a characteristic rough, porous morphology composed of fine, granular clusters [23].

Among these low-temperature samples, the film on the AlN template stands out as an exception, displaying a surface roughness approximately twice that of the others. This unique behavior warrants further investigation in subsequent characterizations.

**Figure 3:**
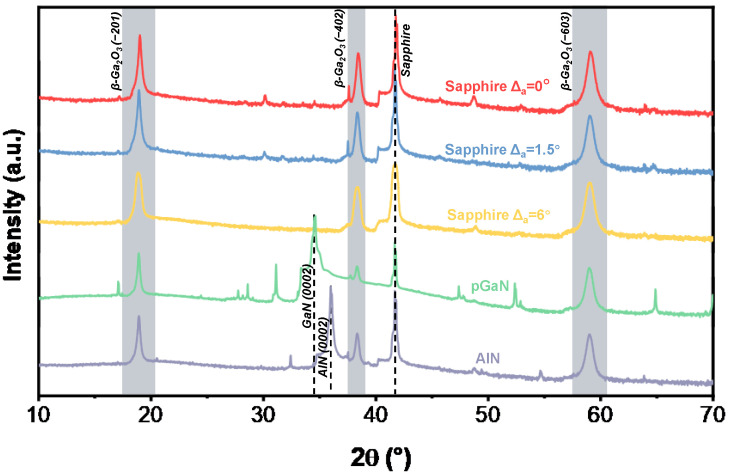
XRD ω-2θ scans of β-Ga_2_O_3_ films grown on different substrates at 800 °C.

Figure 3 presents the XRD ω-2θ scan patterns of the β-Ga_2_O_3_ films grown at 800 °C. The distinct diffraction peaks observed at 18.9°, 38.4°, and 59.1° are assigned to the (−201), (−402), and (−603) planes of β-Ga_2_O_3_, respectively. This series of well-defined peaks confirms the successful heteroepitaxial growth of single-crystal β-Ga_2_O_3_ films with excellent crystalline quality at this temperature. The peak located at 41.7° originates from the sapphire substrate. Additionally, the characteristic peaks of the underlying templates are detected at 34.6° for the pGaN substrate and at 35.9° for the AlN substrate, corresponding to the GaN (002) and AlN (002) reflections, respectively.

A notable finding is the appearance of multiple secondary peaks in the XRD pattern of pGaN substrate. These extraneous peaks are located at 17.0°, 27.7°, 28.6°, 47.4°, 52.3°, and 64.8° [24]. Comparative analysis with standard XRD databases reveals that their presence is primarily attributed to two interrelated factors:

Formation of Polycrystalline β-Ga_2_O_3_: The peaks at 27.7°, 47.4°, and 64.8° are identified as reflections from the (111), (020), and (800) planes of polycrystalline β-Ga_2_O_3_, respectively. Their emergence signifies the nucleation of randomly oriented β-Ga_2_O_3_ grains, which competes with the dominant (−201)-oriented epitaxial growth. This phenomenon is likely driven by the significant lattice mismatch and high interfacial energy between β-Ga_2_O_3_ and the p-GaN template.

Interfacial Reaction and Template Degradation: The peaks at 17.0° and 28.6° suggest complex interfacial reactions. The peak at 17.0° may be indexed to the (10−10) plane of GaN, potentially indicating texture evolution or strain relaxation within the underlying template. The peak at 28.6° does not match any common Ga_2_O_3_ polymorphs. Instead, it is hypothesized to originate from a gallium oxynitride (GaON) transition layer, formed due to the interdiffusion of oxygen and nitrogen atoms at the high-temperature growth interface.

In summary, the coexistence of the dominant (−201) epitaxial phase with polycrystalline β-Ga_2_O_3_ and interfacial reaction products underscores the substantial challenge in achieving phase-pure β-Ga_2_O_3_ epitaxy on p-GaN, stemming from inherent chemical and structural incompatibilities.

**Figure 4:**
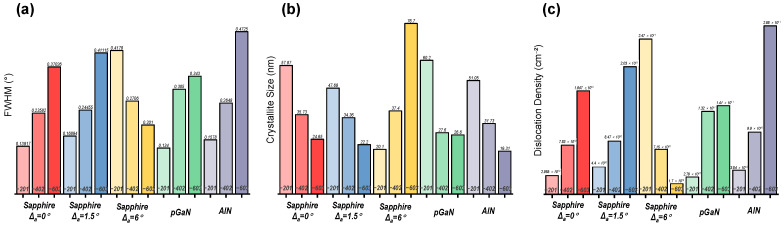
Histograms of (**a**) FWHM, (**b**) crystallite size, and (**c**) dislocation density for different diffraction peaks of β-Ga_2_O_3_ films grown at 800 °C, derived from XRD measurements.

The crystal quality and stress state of the heteroepitaxial β-Ga_2_O_3_ films were systematically analyzed using XRD. Table 1 summarizes the peak positions of the (−201), (−402), and (−603) planes for films grown at 800 °C on different substrates. The deviation of these peaks from the standard β-Ga_2_O_3_ PDF card values (−201: 18.94°, −402: 38.38°, −603: 59.08°) is discussed [25], along with the determined type of stress (compressive or tensile) induced by each substrate. Furthermore, the full width at half maximum (FWHM), crystallite size, and dislocation density, which provide additional insights into the crystalline perfection, are presented in Figure 4.

The Full Width at Half Maximum (FWHM) Calculation for each peak was determined by interpolating the XRD data around the peak and calculating the width at half maximum intensity. The FWHM values were used for subsequent crystallite size and dislocation density calculations.

The crystallite size (*D*) was estimated using the Scherrer equation [26]:

$$D=\frac{K\lambda }{\beta cos\theta }$$

where K=0.9 is the Scherrer constant (shape factor), λ=1.5406 Å is the wavelength of Cu Kα radiation, β is the FWHM in radians (converted from degrees), and θ is the Bragg angle in radians (half of the measured 2θ value).

The dislocation density (*δ*) was calculated using the following formula, which relates to the crystallite size [27]:

$$\delta =\frac{1}{D^{2}}$$

where *D* is the crystallite size in cm. This formula assumes that dislocations are uniformly distributed and that the crystallite size represents the average domain size free of dislocations.

These calculations provide quantitative insights into the film’s microstructure, including stress state, crystallite size, and defect density, which are critical for evaluating the quality of heteroepitaxial growth.

At the growth temperature of 800 °C, the β-Ga_2_O_3_ film grown on the sapphire substrate without an off-cut is the only sample that exhibits compressive stress across all measured crystal planes. This distinct stress state, which differs from that observed in other samples, is likely a primary contributor to its significantly larger surface fluctuations.

For the film on the 6° off-cut sapphire substrate, the full width at half maximum (FWHM) of the (−201), (−402), and (−603) diffraction peaks progressively decreases. This trend is opposite to that observed for the 0° and 1.5° off-cut substrates. This indicates that for the film on the 6° off-cut substrate, the (−201) plane possesses the smallest crystallite size, while the (−603) plane exhibits the lowest dislocation density compared to the other planes. This improved crystallinity with higher diffraction order suggests that the well-defined atomic steps provided by the 6° off-cut surface facilitate superior epitaxial growth, which is responsible for the exceptionally smooth morphology.

It is noteworthy that the FWHM from XRD ω-2θ scans shows an increasing trend with larger off-angle substrate, which is primarily attributed to the increased nucleation density and grain refinement on high-step-density surfaces. However, this does not equate to an overall degradation of crystal quality. Literature [28] reports that in similar systems, films grown on large off-angle substrates exhibit significantly reduced XRD rocking curve FWHM, indicating substantially improved orientation uniformity. This comparison reveals the dual role of off-angle substrate in heteroepitaxy: on one hand, it increases nucleation centers through high-density atomic steps, refining grain size; on the other hand, it forces grains to grow along specific orientations through these ordered steps, reducing mosaic spread. Therefore, the selection of off-angle substrate represents a crucial trade-off between controlling grain size and optimizing crystal orientation.

Regarding the film on the pGaN substrate, its remarkably smooth surface observed by both AFM and SEM can be attributed to the formation of polycrystalline phases. It is postulated that the random nucleation of polycrystalline grains effectively relieved the epitaxial strain. Consequently, a superior surface morphology was achieved at the expense of crystalline phase purity.

**Figure 5:**
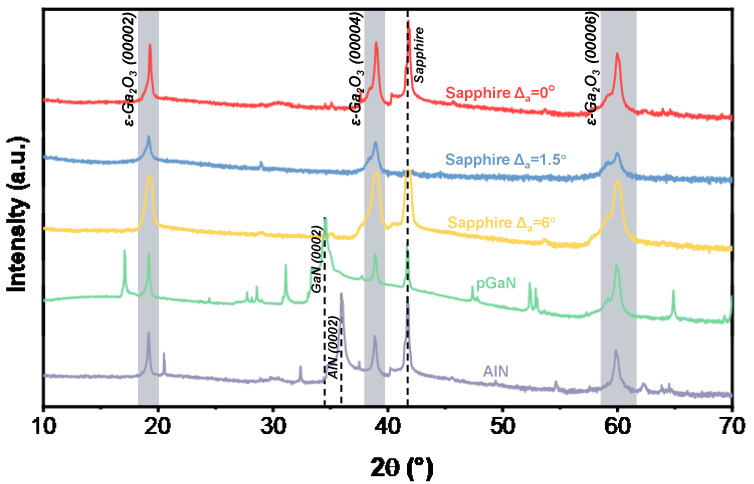
XRD ω-2θ scans of ε-Ga_2_O_3_ films grown on different substrates at 400 °C.

The crystallinity of the ε-Ga_2_O_3_ films was critically dependent on the underlying template. X-ray diffraction (XRD) 2θ-ω scans were employed to assess the phase purity, as shown in Figure 5.

During the growth process at 400 °C, ε-phase gallium oxide was obtained, with its (0002), (0004), and (0006) diffraction peaks located at 19.3°, 39.0°, and 60.0°, respectively. Notably, the (0006) diffraction peak is not symmetric; a shoulder peak approximately at 59.1° is consistently observed on the left side in each sample, indicating the simultaneous presence of β-Ga_2_O_3_.

When ε-Ga_2_O_3_ was heteroepitaxially grown on a p-GaN/Al_2_O_3_ template, the diffraction pattern revealed multiple secondary peaks at 17.07°, 31°, 52.3°, 52.87°, and 64.87°. These peaks are identified as polycrystalline β-Ga_2_O_3_, which is the thermodynamically stable phase. The formation of the β-phase is attributed to the significant lattice mismatch and the instability of the GaN surface under oxidizing growth conditions, which promotes random nucleation of the stable polymorph.

In contrast, growth on an AlN/Al_2_O_3_ template yielded a significant improvement in phase purity. The dominant peaks correspond to the ε-Ga_2_O_3_ phase. However, minor secondary peaks were observed at 20.49° and 32.4°, which are associated with α-Ga_2_O_3_. The emergence of these defects is likely due to non-optimal growth kinetics, as the hexagonal AlN template provides a superior structural match for the metastable ε-phase. This result confirms that AlN is a more suitable template for the heteroepitaxy of pure ε-Ga_2_O_3_, though further process optimization is required to completely suppress competing phases.

**Figure 6:**
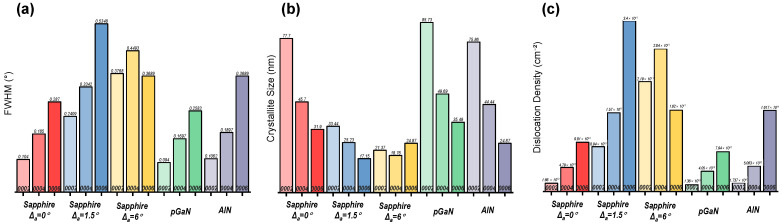
Histograms of (**a**) FWHM, (**b**) crystallite size, and (**c**) dislocation density for different diffraction peaks of ε-Ga_2_O_3_ films grown at 400 °C, derived from XRD measurements.

A systematic analysis of the crystal quality and stress state for the films grown at 400 °C was conducted via XRD. The peak positions for the (0002), (0004), and (0006) planes, corresponding to the ε-Ga_2_O_3_ phase, are detailed in Table 2 for each substrate. The observed shifts in these peaks from the reference values of the ε-Ga_2_O_3_ PDF card (0002: 19.20°, 0004: 38.60°, 0006: 59.40°) are evaluated and attributed to the strain induced by the substrate [29], with the nature of the stress (compressive or tensile) specified. Complementary parameters, including the FWHM, crystallite size, and dislocation density derived from these measurements, are provided in Figure 6.

The XRD peak positions for the 0° and 6° off-cut sapphire substrates consistently shift to higher angles across all measured planes, indicating the presence of compressive stress within the films, which leads to a reduction in interplanar spacing.

In contrast, the films on the 1.5° off-cut sapphire, pGaN, and AlN substrates exhibit a more complex stress state. The (0002) plane shows signs of tensile stress, while the (0004) and (0006) planes indicate compressive stress. This non-uniform stress distribution reveals significant stress anisotropy, likely originating from lattice mismatch or defect formation during heteroepitaxy. For these samples, the crystallite size decreases, and the dislocation density increases with higher-order reflections. This trend suggests that the higher-index planes are more sensitive to microstructural defects. The concomitant increase in FWHM further confirms a gradual degradation of crystalline quality along the c-axis direction. The observed peak shifts from the standard ε-Ga_2_O_3_ positions are attributed to substrate-induced strain and specific growth conditions.

Specifically for the 0°, 1.5° sapphire, pGaN, and AlN substrates, the (0002) plane exhibits the smallest FWHM, the largest crystallite size, and the lowest dislocation density, indicating the highest crystalline perfection near this orientation. The degradation in FWHM, crystallite size, and dislocation density for the (0004) and (0006) planes confirms that crystal quality deteriorates progressively along the c-axis. In summary, while the film growth is preferential along the [0002] direction, significant stress inhomogeneity persists, necessitating further optimization of growth parameters to mitigate stress and reduce dislocation densities.

The calculated crystallite sizes at 400 °C do not show a marked difference from those at 800 °C. This implies that the distinct grain morphologies observed by SEM at these two temperatures are primarily due to differences in the macroscopic aggregation of grains, rather than a fundamental change in the primary crystallite size. This aggregation state is governed by the surface migration energy of adatoms, which is substantially lower at 400 °C. The restricted atomic mobility at the lower temperature results in a “lazier” adatom behavior, inhibiting the reorganization into thermodynamically stable configurations. Conversely, at 800 °C, where migration energy is sufficient, heteroepitaxial β-Ga_2_O_3_ growth is driven to find low-energy sites, which, due to lattice and thermal mismatch with the substrates, paradoxically results in macroscopically rougher surface morphologies.

Furthermore, the notably larger surface roughness observed for the AlN substrate at 400 °C via AFM and SEM could be attributed to the coexistence of multiple Ga_2_O_3_ polymorphs (β, ε, and α phases), introducing additional phase boundaries and mismatch, thereby exacerbating the surface instability.

In general, XRD results indicate that the films on sapphire without an off-cut and on pGaN substrates exhibit relatively narrower FWHM at both 400 °C and 800 °C compared to other substrates. However, the pGaN substrate also presents numerous secondary phases, implying poorer crystalline phase purity. This observation reveals an important discrepancy when correlated with AFM and SEM findings: a smoother surface morphology does not necessarily equate to higher crystalline quality or phase purity. This underscores the necessity for complementary characterization techniques to obtain a comprehensive understanding of the film properties. Further in-depth analysis is required to elucidate the underlying mechanisms.

**Figure 7:**
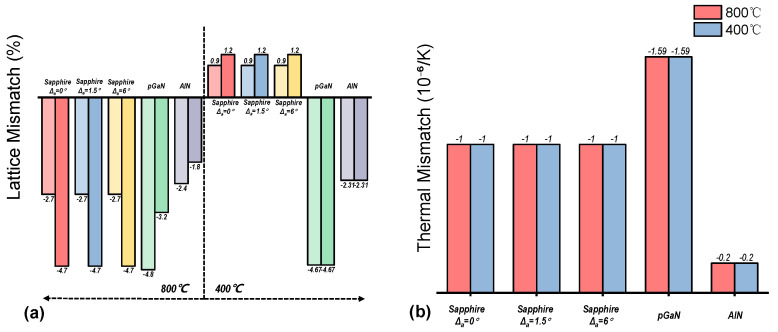
Histograms illustrating the (**a**) lattice mismatch and (**b**) thermal mismatch between the heteroepitaxial gallium oxide films and various substrates.

Figure 7 summarizes the calculated lattice and thermal mismatch for the gallium oxide films grown on various substrates at different temperatures [30].

The lattice mismatch for β-Ga_2_O_3_ and ε-Ga_2_O_3_ on sapphire substrates was calculated based on common epitaxial relationships, such as β-Ga_2_O_3_(−201) || Al_2_O_3_(0001). For β-Ga_2_O_3_ on pGaN(0002) and AlN(0002), the calculation was based on relationships like β-Ga_2_O_3_(−201) || GaN(0001). Since ε-Ga_2_O_3_ possesses a hexagonal structure and exhibits in-plane isotropy, only one lattice mismatch value (along the [11,12,13,14,15,16,17,18,19,20] direction) is listed for it.

The thermal mismatch was derived from the difference in the in-plane coefficients of thermal expansion (CTE) between the substrates and the gallium oxide films. The employed CTE values were approximately 5.0 × 10^−6^/K for sapphire, 4.0 × 10^−6^/K for both β-Ga_2_O_3_ and ε-Ga_2_O_3_, 5.59 × 10^−6^/K for GaN, and 4.2 × 10^−6^/K for AlN. It is noted that the actual mismatch may vary slightly depending on specific growth conditions and film thickness.

Notably, the pGaN substrate exhibits the largest lattice and thermal mismatch with gallium oxide among all substrates at both 800 °C and 400 °C. However, its XRD results show the narrowest FWHM alongside the highest number of secondary phases. In contrast, the AlN substrate demonstrates a relatively smaller thermal mismatch at both temperatures.

**Figure 8:**
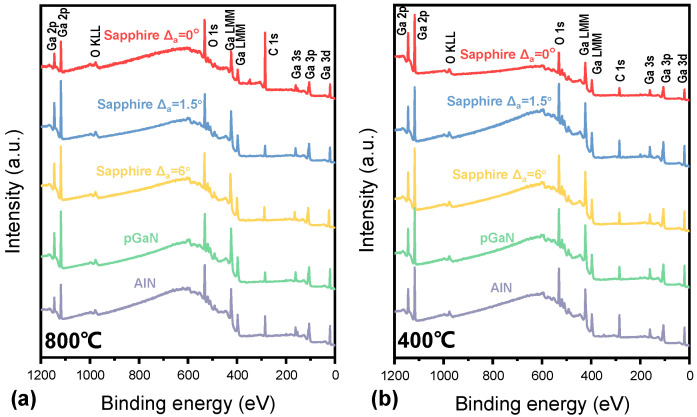
XPS survey spectra (0–1200 eV) of gallium oxide films grown under ten different conditions (combining two temperatures and five substrates): (**a**) 800 °C and (**b**) 400 °C.

The survey XPS spectra of the films grown on different substrates at 800 °C and 400 °C are presented in Figure 8. Distinct peaks corresponding to gallium (Ga) and oxygen (O) confirm the formation of gallium oxide films. Subsequent high-resolution analysis of the O 1s peak was performed, with the spectra deconvoluted into at least two components: a low-binding-energy component (~530.0–530.6 eV), attributed to the Ga–O bonds (lattice oxygen) in β-Ga_2_O_3_, and a high-binding-energy component (~531.2–532.0 eV), ascribed to surface species such as hydroxyl groups (-OH), adsorbed water (H_2_O), or carbonates [31]. The percentage of lattice oxygen and the ratio of adsorbed oxygen to lattice oxygen were extracted from this analysis and are displayed in this study.

**Figure 9:**
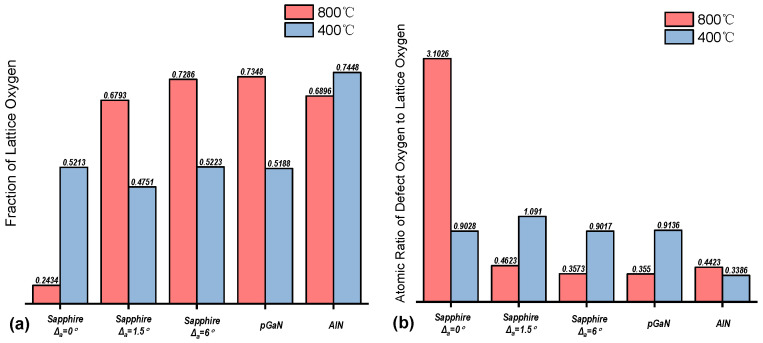
Histograms of the relative percentages of (**a**) the fraction of lattice oxygen and (**b**) the atomic ratio of defective oxygen to lattice oxygen in the O 1s region for films grown under 800 °C and 400 °C, obtained from peak deconvolution of high-resolution XPS spectra.

Figure 9 summarizes the quantitative results, showing the lattice oxygen percentage and the adsorbed-oxygen-to-lattice-oxygen ratio. For the film grown at 800 °C on the sapphire substrate without an off-cut, the O 1s spectrum exhibits a lower lattice oxygen percentage and a higher adsorbed-oxygen-to-lattice-oxygen ratio. This indicates a higher density of surface defects, which is consistent with its inferior surface morphology observed by AFM/SEM. Meanwhile, samples grown at 400 °C generally show a higher proportion of adsorbed oxygen. The significantly higher proportion of adsorbed oxygen species in the XPS O 1s spectra of the 400 °C samples indicates a higher density of surface sites with high reactivity.

**Figure 10:**
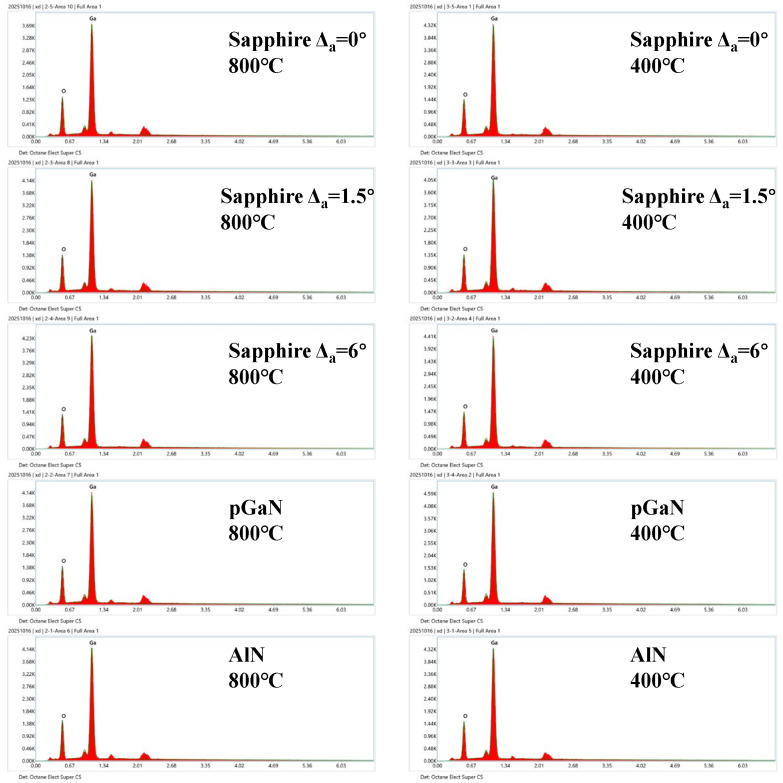
Elemental mapping results from energy-dispersive X-ray (EDX) spectroscopy for gallium oxide films grown under ten different conditions (combining two temperatures and five substrates).

The energy-dispersive X-ray (EDX) spectroscopy mapping (10 kV accelerating voltage) results are presented in Figure 10, which provides the elemental composition and spatial distribution of Ga and O. A quantitative analysis of the O/Ga atomic ratio was performed, and the results are compared with those from XPS.

**Figure 11:**
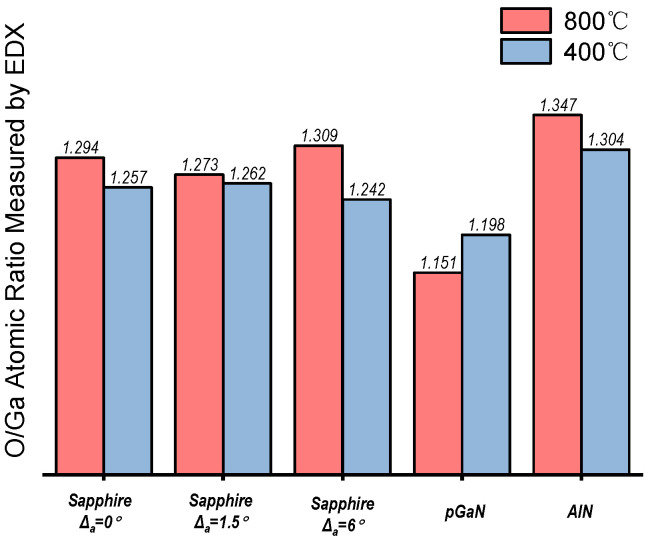
Histogram of O/Ga atomic ratios derived from EDX analysis for gallium oxide films grown under ten different conditions.

Figure 11 displays a statistical graph of the O/Ga atomic ratio for all samples, offering an intuitive visualization of the oxygen deficiency and its correlation with both growth temperature and substrate type. The films grown at 800 °C exhibit a higher O/Ga atomic ratio compared to those grown at 400 °C, indicating a composition closer to the ideal stoichiometric value of 3:2 and thus higher quality gallium oxide films [21]. Among the different substrates at 800 °C, the AlN substrate yields the best result (closest to stoichiometry), while the pGaN substrate gives the poorest. This trend aligns with the lattice and thermal mismatch data presented earlier: pGaN has the largest mismatch with Ga_2_O_3_, while AlN has a relatively smaller mismatch. For the sapphire substrates, the film on the 6° off-cut substrate shows a superior O/Ga ratio at 800 °C, whereas the 1.5° off-cut substrate is more favorable at 400 °C.

In summary, the samples grown at 800 °C are more stoichiometric than those grown at 400 °C, implying more sufficient incorporation and reaction between Ga and O precursors at the elevated temperature. Furthermore, the oxygen incorporation efficiency varies significantly across different substrates. The superior performance of the AlN substrate can be linked to its high chemical stability. In contrast, the poor result for the pGaN substrate may be attributed to its pronounced lattice/thermal mismatch and the potential thermal decomposition of the underlying GaN template, which could detrimentally affect oxygen incorporation during growth.

**Figure 12:**
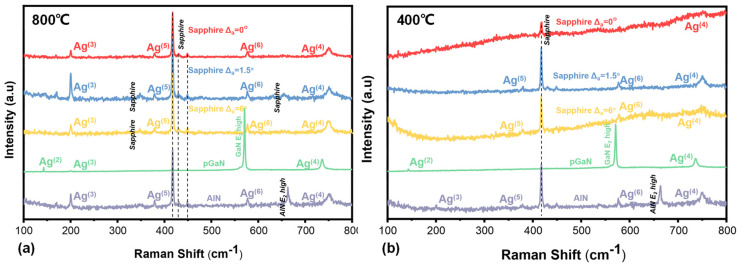
Raman spectra of gallium oxide films grown under ten different conditions: (**a**) 800 °C and (**b**) 400 °C.

Figure 12 presents Raman spectra of β-Ga_2_O_3_ heteroepitaxial films grown at 800 °C and 400 °C on various substrates. The characteristic phonon modes for both the epitaxial β-Ga_2_O_3_ films and the underlying substrates are identified and labeled in the figure. The observed peak positions and their assignments are summarized in Table 3. The data conclusively confirm the successful heteroepitaxial growth of β-Ga_2_O_3_, as evidenced by the presence of its characteristic Ag and Bg modes [32]. The detection of substrate-related peaks (from sapphire, GaN, and AlN) confirms the sufficient penetration depth of the 532 nm laser excitation source.

At the growth temperature of 800 °C, the characteristic Raman peak is consistently observed at 200.57 cm^−1^ for films on all five substrates. This uniformity in peak position indicates an effective release of intrinsic stress within the epitaxial films. The corresponding full width at half maximum (FWHM) values for the five substrates are 2.5, 2.53, 3.49, 2.3, and 2.33 cm^−1^, respectively.

In contrast, at the lower temperature of 400 °C, the Ag^(3)^ phonon mode is only detectable in the film grown on the AlN substrate. The absence of this characteristic peak in other samples can be primarily attributed to their poorer overall crystalline quality. Furthermore, the Raman spectrum acquired from the AlN sample exhibits a distinctive hump-shaped background. This feature is likely associated with fluorescence effects occurring at the film surface, which can interfere with the Raman signal.

## 4. Conclusions

In summary, this study identifies 800 °C as the optimal growth temperature for high-quality β-Ga_2_O_3_ heteroepitaxy, yielding superior crystallinity, improved stoichiometry (closer to the ideal O/Ga ratio of 3:2), and a reduced oxygen deficiency. The 6° off-cut sapphire and AlN/sapphire substrates are the most favorable, effectively mitigating non-stoichiometry and defect formation. A key finding is that surface smoothness can be misleading, as it does not guarantee high phase purity or controlled oxygen deficiency, exemplified by the p-GaN substrate. These results provide clear guidelines for optimizing chemical composition and defect control in β-Ga_2_O_3_ device fabrication.

## Figures and Tables

**Table 1:** XRD Peak Positions and Stress State Results for Gallium Oxide Heteroepitaxial Films at 800 °C.

Substrate	hkl	Measured Peak Position (°2θ)	Peak Shift (°2θ)	Stress State
Sapphire Δ_a_ = 0°	−201	18.9996	0.0596	Compressive
	−402	38.4209	0.0409	Compressive
	−603	59.0888	0.0088	Compressive
Sapphire Δ_a_ = 1.5°	−201	18.9022	−0.0378	Tensile
	−402	38.343	−0.037	Tensile
	−603	59.0304	−0.0496	Tensile
Sapphire Δ_a_ = 6°	−201	18.8438	−0.0962	Tensile
	−402	38.343	−0.037	Tensile
	−603	59.0499	−0.0301	Tensile
pGaN	−201	18.9	−0.04	Tensile
	−402	38.32	−0.06	Tensile
	−603	59.01	−0.07	Tensile
AlN	−201	18.9217	−0.0183	Tensile
	−402	38.343	−0.037	Tensile
	−603	59.0109	−0.0691	Tensile

**Table 2:** XRD Peak Positions and Stress State Results for Gallium Oxide Heteroepitaxial Films at 400 °C.

Substrate	hkl	Measured Peak Position (°2θ)	Peak Shift (°2θ)	Stress State
Sapphire Δ_a_ = 0°	0002	19.27	0.07	Compressive
	0004	38.99	0.39	Compressive
	0006	59.98	0.58	Compressive
Sapphire Δ_a_ = 1.5°	0002	19.175	−0.025	Tensile
	0004	38.927	0.327	Compressive
	0006	59.965	0.565	Compressive
Sapphire Δ_a_ = 6°	0002	19.2334	0.0334	Compressive
	0004	38.9858	0.3858	Compressive
	0006	60.0433	0.6433	Compressive
pGaN	0002	19.1749	−0.0251	Tensile
	0004	38.9079	0.3079	Compressive
	0006	59.9265	0.5265	Compressive
AlN	0002	19.136	−0.064	Tensile
	0004	38.849	0.249	Compressive
	0006	60.004	0.604	Compressive

**Table 3:** Raman Peak Position Results for Gallium Oxide Heteroepitaxial Films.

Observed Peak (cm^−1^)	Assigned Material	Phonon Mode
β-Ga_2_O_3_		
~142–143	β-Ga_2_O_3_	Ag^(2)^
~200.5	β-Ga_2_O_3_	Ag^(3)^
~379.3	β-Ga_2_O_3_	Ag^(5)^
~577.4	β-Ga_2_O_3_	Ag^(6)^
~736.2	β-Ga_2_O_3_	Ag^(4)^
~750.7	β-Ga_2_O_3_	Ag^(4)^
Sapphire (α-Al_2_O_3_)		
~347.4	Sapphire	Eg
~417.6	Sapphire	A_1_g
~429.6	Sapphire	Eg
~449.0	Sapphire	Eg
~654.6	Sapphire	Eg
GaN/AlN		
~570.9	GaN	E_2_ (high)
~663.0	AlN	E_2_ (high)

## Data Availability

The original contributions presented in this study are included in the article. Further inquiries can be directed to the corresponding author(s).
